# Effective Strategies to Reduce Anxiety in Patients Undergoing Cataract Surgery: An Umbrella Review

**DOI:** 10.1002/hsr2.72637

**Published:** 2026-06-07

**Authors:** Masoumeh Masoumy, Shahnaz Ahrari, Mehdi Rezaei

**Affiliations:** ^1^ Department of Operation Room, Faculty of Allied Medical Sciences Bushehr University of Medical Sciences Bushehr Iran; ^2^ Nursing and Midwifery School, Faculty of Nursing and Midwifery Birjand University of Medical Sciences Birjand Iran; ^3^ Department of Psychology, Faculty of Educational Science and Psychology University of Birjand Birjand Iran

**Keywords:** anxiety, cataract, preoperative care, surgery, systematic review

## Abstract

**Introduction:**

Anxiety, which involves feelings of tension, worry, and physiological changes in the body, can have significant impacts on patients, including an increased risk of mortality. In ophthalmic surgeries, particularly cataract procedures, anxiety levels tend to be high, often stemming from fears of blindness or surgical failure. This study aimed to determine the best and most effective interventions to reduce anxiety in patients undergoing cataract surgery.

**Methods:**

Systematic reviews, with or without meta‐analysis. Additionally, selected studies were required to meet two mandatory criteria from the Database of Abstracts of Reviewers of Effects and be English‐language review articles published between January 2010 and 2025 that met these criteria and focused on anxiety reduction strategies in patients undergoing cataract surgery.

**Results:**

Finally, out of 75 relevant papers, 5 review studies with 9638 patients were eligible and included in the study. (1) Non‐pharmacological interventions (educational videos, patient education, aromatherapy, relaxation techniques, etc.) significantly reduced mean preoperative anxiety compared to the control group. (SMD: −2.14, 95% CI: −3.48 to −0.79; *p* < 0.001). (2) Nursing techniques could reduce pain and anxiety during the operation (SMD = − 1.19; 95% (CI): −1.96 to −0.43; *p* = 0.002). (3) The use of anxiolytics (melatonin) could reduce postoperative anxiety in cataract patients. (SMD = − 0.55; 95% CI: −0.95 to −0.15; *p* = 0.007). (4) Music therapy.

**Conclusion:**

This review study identified techniques and strategies to reduce stress in patients undergoing cataract surgery. These strategies, tailored to patient needs, can be implemented individually or in combination, and prioritizing individual patient needs to enhance patient well‐being and lead to several positive clinical outcomes and potentially decrease healthcare costs. Future clinical trials are essential to the integration of new technologies and identifying the most effective methods for widespread implementation.

## 1 Introduction

Cataracts are the most common treatable cause of blindness in older people, affecting approximately 11.8%–18.8% of individuals over 40 [1]. Globally, over 10 million cataract surgeries are performed annually, a number that continues to rise [2]. Recent advances in anesthesia and surgical methods have enabled most cataract surgeries to be performed using local anesthesia. This progress has significantly shortened operating times and minimized the side effects related to both local and general anesthesia. However, despite the use of sedatives such as benzodiazepines and opioids administered before and during the procedure, patients may still experience considerable anxiety, as well as some pain and discomfort throughout the procedure [3]. Anxiety, which involves feelings of tension, worry, and physiological changes in the body, can have significant impacts on patients, including an increased risk of mortality [4, 5, 6, 7]. In ophthalmic surgeries, particularly cataract procedures, anxiety levels tend to be high, often stemming from fears of blindness or surgical failure [8]. The sources of anxiety related to cataract surgery are multifaceted, with preoperative uncertainty, fear of potential complications, and concerns about the surgical outcome playing major roles in elevating patients' anxiety levels [9]. Numerous studies have demonstrated that elevated preoperative anxiety is associated with a range of adverse outcomes, including increased pain perception, elevated requirement for anesthetic medications, and longer recovery times. Additionally, high anxiety levels are associated with serious complications such as postoperative delirium and cardiac events [10]. Given its significant impact on surgical success and patient satisfaction, it is crucial to identify evidence‐based strategies to reduce preoperative anxiety. Recent research highlights various approaches, including preoperative education, pharmacological interventions, and cognitive‐behavioral therapies, which have shown promise in reducing anxiety levels among patients [11].

Both pharmacological and non‐pharmacological methods are available for alleviating preoperative anxiety. Since non‐pharmacological techniques generally have fewer side effects and can be applied across different age groups and conditions, there is an increasing need to thoroughly evaluate their effectiveness in cataract surgery and their role in reducing preoperative anxiety [8]. With the growing number of systematic reviews in this field, clinicians and policymakers face the challenge of synthesizing extensive information. Consequently, conducting umbrella reviews—comprehensive analyses that integrate and summarize findings from multiple reviews—becomes an essential step to provide clear and evidence‐based guidance for healthcare decision‐makers [12].

This umbrella review aimed to gather and synthesize existing evidence on these strategies, offering healthcare providers a clear and practical overview to effectively manage patient perioperative anxiety. This review was designed to identify the most effective strategies for reducing anxiety in patients undergoing cataract surgery.

## 2 Methods

This umbrella review was conducted according to the Preferred Reporting Items for Systematic Reviews and Meta‐Analysis (PRISMA 2020) protocol [13].

### 2.1 Research Questions

What are effective interventions to reduce anxiety in patients undergoing cataract surgery?

### 2.2 Inclusion and Exclusion Criteria

Following standard practices in evidence analysis, inclusion criteria were determined using the PICOS framework:

**Population:** Inclusion: patients undergoing cataract surgery. Exclusion: studies not focused on patients undergoing cataract surgery. **Intervention:** Inclusion: interventions proven effective in reducing anxiety among patients undergoing cataract surgery, including those conducted in hospitals, outpatient settings, or private centers. Exclusion: studies that did not include the relevant intervention. **Comparison:** Inclusion: studies demonstrating increased adherence to guidelines related to anxiety reduction in patients undergoing cataract surgery. Exclusion: reviews that included non‐clinical trial studies or qualitative research. **Outcome:** Inclusion: improved adherence to anxiety reduction guidelines among patients undergoing cataract surgery. Exclusion: studies not reporting relevant outcomes. **Study design:** Inclusion: systematic reviews, meta‐analyses, and reviews with various types of clinical trials, including those that meet the two mandatory criteria of the Database of Abstracts of Reviews of Effects (DARE) [12, 14]. Finally, we included only English‐language review articles published between January 2010 and 2025 that met these criteria and focused on anxiety reduction strategies in patients undergoing cataract surgery. Abstracts, preliminary reports, and outdated or withdrawn theses were excluded from the analysis.

### 2.3 Search Strategy

A comprehensive systematic search was conducted from January 2010 to 2025 by two independent, trained researchers (S.A., M.M., and M.R.) across multiple databases, including PubMed, Scopus, Web of Science, ProQuest, Google Scholar, Medline, and Science Direct. The search employed keywords derived from MeSH terms and relevant literature, such as *Preoperative care*, *surgery*, *anxiety*, *cataract,* and *systematic review*. The operators “NOT” and “AND” were used appropriately to refine the search.

### 2.4 Screening, Data Extraction, and Quality Assessment (Risk of Bias)

Initially, titles, abstracts, and full texts of articles were reviewed independently by two researchers using EndNote. For article selection, two reviewers (S.A. and M.M.) assessed both the full texts and abstracts for eligibility according to the PRISMA (2020) guidelines. Any disagreements were resolved through discussion between the reviewers. If consensus could not be reached, a third reviewer (M.R.) was consulted for resolution.

The AMSTAR2 was employed to evaluate the methodological quality of the included systematic reviews. AMSTAR2 consists of 16 items covering various aspects of review quality, including the use of PICO, registration of the review protocol, inclusion criteria, search strategy, data extraction, reporting of included and excluded studies, assessment of methodological quality, funding, meta‐analysis methods, heterogeneity, and publication bias. Each item is answered with “Yes,” “No,” “Cannot answer,” or “Not applicable,” with only “Yes” responses earning points. The maximum attainable score is 16. Reviews in which fewer than 40% of the items are marked “Yes” are considered as low quality. Those with 40%–80% “Yes” responses are considered as moderate quality. Reviews achieving at least 80% “Yes” responses are considered high quality [12, 15].

### 2.5 Data Extraction

The included review articles were distributed among the study authors, and two researchers (S.A. and M.M.) extracted data independently. They manually reviewed each article to extract key information such as search databases, study objective, sample size, intervention type, and main results. Subsequently, strategies suggested for improving physical activity adherence were compiled through a careful review of the results, discussion, and conclusion sections of each article. These extracted strategies were identified to positively influence physical activity adherence in the target populations. The extracted data were then compared between the two researchers, and any discrepancies were reviewed and resolved. Any remaining disagreements were resolved by consulting a third researcher (M.A.) to ensure the accuracy and validity of the extracted data.

### 2.6 Data Synthesis

A meta‐analysis was not feasible due to heterogeneity in interventions and outcome measures. Therefore, a narrative synthesis was conducted, presenting extracted data in summary Table 3. The first reviewer (S.A.) prepared the initial synthesis, and the second, third, and fourth reviewers (S.A./M.M. and R.J.) reviewed it. Discrepancies were resolved through consensus, with the fifth referee (S.A.) making the final decision when necessary.

## 3 Results

**Figure 1:**
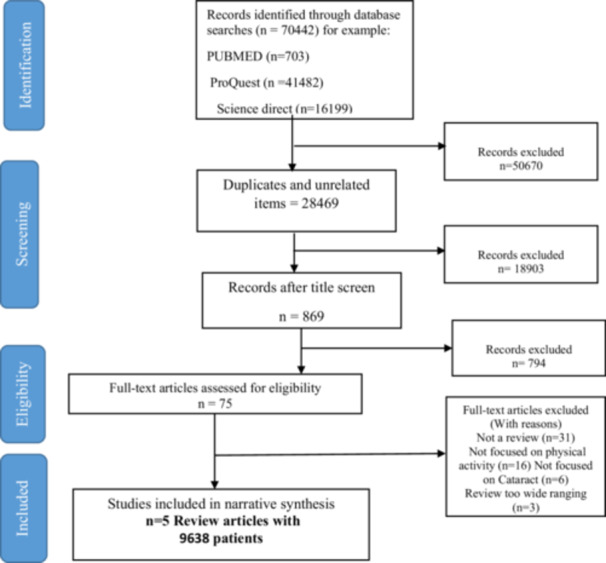
The PRISMA flow diagram of selected studies.

The number of papers and the method of selection based on PRISMA (2020) are provided in Figure 1.

Table 1 indicates the results of this quality assessment. Table 2 indicates the characteristics of the included review studies and their sample. Table 3 indicates a summary of studies presenting the strategies for physical activity adherence in patients with chronic obstructive pulmonary disease.

**Table 1:** Characteristics of included review studies and their sample.

Study	Country	Population (M/F)	Mean age (year)	Type of studies in reviews	Number of patients	Number of Studies
Nabighadim et al. 2025 [8]	Iran	‐‐	≤ 18 up to > 60 years	Systematic review and meta‐analysis	1998	22
Dahshan et al. 2021[16]	China	‐‐	from 50 to 89 years	Systematic review	523	3
Baek et al.2024 [17]	Belgium	‐‐	from 65.8 to 74	Integrative review	1641	12
Zeng et al. 2021 [18]	China	‐‐	66.2	Systematic review and meta‐analysis	3378	19
Chen et al. 2024 [19]	China	70.86% M	63.5	Systematic review and meta‐analysis	2098	15

**Table 2:** Result of the quality assessment of reviews using the AMSTAR 2 checklist.

AMSTAR 2 items																	
Study	Q1	Q2	Q3	Q4	Q5	Q6	Q7	Q8	Q9	Q10	Q11	Q12	Q13	Q14	Q15	Q16	Quality of article
Nabighadim et al. 2025 [8]	1	1	1	1	1	1	1	1	1	0	1	1	1	1	1	1	94%HQ
Dahshan et al. 2021 [16]	1	0	1	1	1	1	1	1	1	0	n/a	n/a	1	1	n/a	1	69%HQ
Baek et al. 2024 [17]	1	0	1	1	1	1	1	1	1	0	n/a	n/a	1	1	n/a	1	69%HQ
Zeng et al. 2021 [18]	1	0	1	1	1	1	1	1	1	0	1	1	1	1	0	1	81%HQ
Chenet al. 2024 [19]	1	1	1	1	1	1	1	1	1	1	1	1	1	1	1	0	94%HQ

**Table 3:** A summary of studies that offer strategies for reducing anxiety in patients undergoing cataract surgery

Author	Study numbers and publication year	Objective	Sample size	Intervention type	Main results	Type of review
Nabighadim et al. 2025 [8]	*N* = 22 (inception to 2024)	To examine the effects of non‐pharmacological interventions before cataract surgery on preoperative anxiety.	1998 participants	Back massage, hand massage, foot massage, music, educational videos, patient education, and aromatherapy and relaxation techniques.	Non‐pharmacological interventions significantly reduced mean preoperative anxiety compared to the control group. (SMD: −2.14, 95% CI: −3.48 to −0.79; *p* < 0.001) Subgroup analysis showed that all interventions were effective in reducing preoperative anxiety. However, hand and foot massage did not have a significant effect.	Systematic review and meta‐analysis
Dahshan et al. 2021 [16]	*N* = 3 (2000–2021)	To examine the effects of music on patients undergoing cataract surgery.	523 participants	Music therapy	The use of music during cataract surgery was effective in reducing perceived pain and anxiety, and improving vital signs (decreased heart rate and blood pressure).	Systematic review
Baek et al. 2024 [17]	*N* = 12 (2013– 2023)	To identify the effect of non‐pharmacological interventions for preoperative anxiety management among older patients undergoing cataract surgery.	1641 participants	Music therapy, video or multimedia education, massage therapy, and fasting	Non‐pharmacological interventions (music, education, and hand massage) effectively reduced preoperative anxiety levels among older patients undergoing cataract surgery.	Integrative review
Zeng et al. 2021 [18]	*N* = 19 (2000– 2021)	To improve the comfort of cataract surgery patients.	3378 participants	Nursing techniques (audio‐visual training before surgery, limb or back massage, listening to soothing music), anxiolytics, and local anesthetics.	The use of high‐quality nursing techniques could reduce pain and anxiety during the operation (SMD = − 1.19; 95% (CI): −1.96 to −0.43; *p* = 0.002). The use of anxiolytics (melatonin) could reduce postoperative anxiety in cataract patients.(SMD = − 0.55; 95% CI: −0.95 to −0.15; *p* = 0.007)	Systematic review and meta‐analysis
Chen et al. 2024 [19]	*N* = 15	To evaluate the effectiveness of music therapy in reducing anxiety, pain, and vital sign changes in ophthalmic surgery patients.	2098 participants	Patients who underwent various ophthalmic surgeries, the intervention group, patients who were exposed to music through headphones or speakers in the operating room.	Music therapy effectively reduced anxiety and pain and moderately improved vital signs of patients undergoing ophthalmic surgery, highlighting its role in enhancing patient well‐being. More in‐depth RCTs are needed to confirm its effectiveness. Targeted interventions improved patients' understanding of post‐cataract surgery care.	Systematic review and meta‐analysis

Finally, out of 69 relevant papers, 5 systematic reviews and meta‐analysis studies with 9638 patients were eligible and included in the study. (1) Non‐pharmacological interventions (educational videos, patient education, aromatherapy, and relaxation techniques, etc.) significantly reduced mean preoperative anxiety compared to the control group. (SMD: −2.14, 95% CI: −3.48 to −0.79; *p* < 0.001). (2) Nursing techniques could reduce pain and anxiety during the operation (SMD = − 1.19; 95% (CI): −1.96 to −0.43; *p* = 0.002). The use of anxiolytics (melatonin) could reduce postoperative anxiety in cataract patients. (SMD = − 0.55; 95% CI: −0.95 to −0.15; *p* = 0.007). (3) Music therapy: The use of music during cataract surgery was effective in reducing perceived pain and anxiety, and improving vital signs (decreased heart rate and blood pressure).

The review by Dahshan et al. showed that playing music during cataract surgery can effectively reduce patients' pain and anxiety, while also improving vital signs such as systolic blood pressure and heart rate [16]. Other reviews similarly indicated that music therapy decreased anxiety and pain in patients undergoing cataract surgery [8, 16, 17]. Music is a low‐risk, cost‐effective, non‐pharmacological alternative to anxiolytic medications [16]. It has been shown to effectively reduce anxiety and pain and to moderately improve vital signs during ophthalmic surgery, thereby contributing to increased patient well‐being. However, more rigorous RCTs are necessary to confirm its efficacy [19].

Furthermore, providing comprehensive information to patients about the cataract procedure has been shown to reduce preoperative anxiety [8, 17]. A review by Zeng et al. also demonstrated that preoperative audio‐visual education diminished both pain and anxiety levels in patients undergoing cataract surgery. The use of high‐quality nursing techniques could reduce pain and anxiety during the operation

(SMD = − 1.19; 95% (CI): −1.96 to −0.43; *p* = 0.002). The use of anxiolytics (melatonin) could reduce postoperative anxiety in cataract patients (SMD = − 0.55; 95% CI: −0.95) [18]. Additionally, massage techniques targeting the limbs and back enhanced blood circulation, decreased muscle spasms, and reduced anxiety before and after cataract surgery [8, 17, 18].

During the postoperative phase, high‐quality nursing interventions—such as preoperative audio‐visual education, limb or back massage, and listening to soothing music—can help reduce pain and anxiety. The use of preoperative anxiolytic agents and sedatives can also alleviate anxiety and improve comfort, particularly in patients with high anxiety levels. The choice of topical anesthetic used during surgery, however, shows no clear benefit in terms of improving patient comfort [18].

A meta‐analysis using a random‐effects model, including 22 studies with 1998 participants, found that various non‐pharmacological interventions—such as back massage, hand massage, foot massage, music, educational videos, patient education, aromatherapy techniques, and relaxation—significantly lowered preoperative anxiety compared to control groups. Subgroup analysis indicated that all these interventions were effective, except for hand and foot massage, which did not show a significant impact. Meta‐regression analysis revealed a significant association between the proportion of women and the effect size. Sensitivity analysis confirmed the robustness of the findings related to non‐pharmacological interventions, while the Knapp–Hartung method preserved the overall effect size but produced wider confidence intervals. Additional high‐quality research is necessary to validate these interventions and establish clearer guidelines [8].

Results of this meta‐analysis showed the GRADE assessment downgraded for preoperative anxiety evidence by one level due to risk of bias from detection bias, one level for inconsistency with an *I* ^2^ of 99.4%, and one level for suspected publication bias. Given the low certainty of the evidence, we can only suggest the potential benefits of hand massage and music, but cannot strongly recommend them. The GRADE assessment downgraded this evidence by one level due to risk of bias from detection, performance, and allocation concealment bias, as well as some inconsistency in the results across studies. Further high‐quality research is needed to establish their effectiveness.

Due to the very low certainty of the evidence, we can only offer tentative recommendations regarding the use of aromatherapy, foot massage, back massage, visual education, relaxation techniques, and educational interventions to reduce preoperative anxiety. The GRADE assessment downgraded this evidence by one level for risk of bias and inconsistency and an additional one to two levels for imprecision due to small sample sizes, leading to wide confidence intervals and less precise effect estimates. However, we cannot confidently recommend their routine use based on the current evidence [8].

Postoperatively, employing high‐quality nursing techniques—such as preoperative audio‐visual education, limb or back massage, and listening to soothing music—can help reduce pain and anxiety. The use of preoperative anxiolytic medications and sedatives can also alleviate anxiety and enhance comfort, particularly in patients experiencing high levels of preoperative anxiety. However, the choice of topical anesthetic during the procedure appears to have no clear advantage in improving patient comfort [18].

These preventive approaches (such as music, nursing techniques, etc.) not only improve patient comfort but can also lead to several positive clinical outcomes. Improving preoperative anxiety management can lead to better surgical outcomes, because patients who experience less anxiety typically have lower postoperative complication rates and shorter recovery times, which can lead to shorter hospital stays and ultimately reduced healthcare costs [8, 17, 20].

## 4 Discussion

Managing anxiety and pain associated with cataract surgery is crucial for significantly enhancing patient comfort and compliance [21]. Ahmed et al. demonstrated that providing educational videos before cataract surgery is a cost‐effective and efficient intervention that significantly reduces patient anxiety and improves their overall experience [22]. These findings emphasize the importance of using preoperative educational tools, which can positively influence both psychological and physiological outcomes. Such approaches not only promote patient well‐being but also enhance cooperation with the healthcare team. Furthermore, Shen et al. highlighted the importance of the teach‐back method in managing anxiety and optimizing patient cooperation during surgery. This method not only reduces anxiety and increases health knowledge, but also impacts stress responses in older patients. The observed decreases in blood pressure and heart rate fluctuations further indicate that the teach‐back method positively affects physiological parameters during the surgical procedure [23].

The study by Cavdar et al. highlighted that hand massage, a simple and cost‐effective technique, not only reduces patients' anxiety, but also positively influences their vital signs and overall comfort. These results underscore the value of incorporating physical and non‐pharmacological methods into the management of patient anxiety [24]. Similarly, Zhang et al. emphasized the significance of integrating video supplementation into the traditional informed consent process. This educational intervention enhances patients' perception of the surgical procedure and reduces anxiety on the day of surgery, which can subsequently improve patient cooperation with the healthcare team [25]. Additionally, Guerrier et al. demonstrated that a personalized web‐based music intervention not only reduces anxiety and hypertension but also decreases the need for sedatives. This innovative, technology‐driven approach holds significant promise for enhancing patient well‐being [26].

Numerous studies have shown that non‐pharmacological interventions play a crucial role in decreasing anxiety and pain in patients undergoing cataract surgery [3]. These findings highlight the importance of using non‐pharmacological and cost‐effective strategies in managing patient anxiety and pain. Incorporating such methods into surgical protocols can enhance the patient experience and potentially improve clinical outcomes. The study by Loong et al. demonstrated that binaural beats, an innovative technique, significantly decrease pain and anxiety in patients undergoing local anesthesia. Additionally, this method can modulate the tachycardia response to stress, contributing to improved physiological stability [27]. Mohammadpourhodki et al. highlighted that gentle lumbar massage, a low‐cost and safe intervention, effectively reduces anxiety in patients undergoing cataract surgery. This simple yet effective approach has considerable potential to enhance patient well‐being [28]. Similarly, Farahani et al. found that hand or foot massage can efficiently alleviate anxiety in patients awaiting phacoemulsification cataract surgery, promoting greater relaxation [29]. Moreover, Dastan et al. emphasized that preoperative hand massage not only reduces anxiety and fear of surgery but also lowers pain levels and positively influences physiological parameters [30].

The review by Park et al. suggested that technology‐based music interventions can effectively diminish anxiety and pain during surgery. These findings provide valuable guidance for healthcare teams in selecting practical music intervention methods. Future research should explore the potential of advanced, technology‐driven music interventions using smart devices and software to facilitate better interaction between healthcare workers and patients [31].

A 2024 meta‐analysis suggested that multimedia educational programs provided before surgery may beneficially impact anxiety levels in women undergoing cesarean sections. However, validating these findings requires larger samples and multicenter randomized controlled trials [32]. This study revealed that watching an informational video before an elective cesarean section reduced preoperative anxiety; however, this reduction was not statistically significant and showed high inconsistency across results. Nevertheless, the intervention did improve women's postoperative satisfaction. Future research with improved methodologies is needed to determine the optimal duration and content for such informational videos [33].

Li et al. indicated that postoperative music therapy can significantly reduce postoperative pain and anxiety, and prevent fluctuations in blood pressure and heart rate. However, it did not enhance patient satisfaction with the hospital or reduce the incidence of postoperative nausea and vomiting [34]. Our meta‐analysis suggests that acupressure can alleviate anxiety, with finger massage proving more effective for inpatients and preoperative patients. Acupressure may be a promising treatment option for individuals with a high level of anxiety and stable hemodynamic status. Nevertheless, due to considerable heterogeneity across studies, any conclusions drawn from these results should be interpreted with caution [35].

## 5 Conclusion

Various studies consistently demonstrated that effective management of anxiety and pain—before, during, and after surgery is crucial for enhancing patient experience and improving clinical outcomes. Non‐pharmacological interventions, including massage, music therapy, and preoperative educational videos, not only reduce anxiety and pain but also positively influence patients' physiological responses and promote greater cooperation throughout the surgical procedure. The integration of new technologies, such as advanced music applications and multimedia educational programs, further facilitates health knowledge dissemination and fosters improved interaction between patients and healthcare workers. These cost‐effective and practical interventions can be readily incorporated into surgical protocols to enhance patient well‐being and potentially decrease the reliance on sedatives. Future large‐scale studies and clinical trials are essential to validate these findings and identify the most effective methods for widespread implementation.

## Author Contributions

**Masoumeh Masoumy:** conceptualization, resource, validation, methodology, visualization, data curation, investigation, writing – original draft. **Shahnaz Ahrari:** conceptualization, investigation, project administration, resources, supervision, validation, visualization, writing – review and editing. **Mehdi Rezaei:** conceptualization, resources, writing – review and editing, validation.

## Funding

The authors received no specific funding for this study.

## Ethics Statement

The authors have nothing to report.

## Conflicts of Interest

The authors declare no conflicts of interest.

## Transparency Statement

The corresponding author, Shahnaz Ahrari, affirms that this manuscript is an honest, accurate, and transparent account of the study being reported; that no important aspects of the study have been omitted and that any discrepancies from the study as planned (and, if relevant, registered) have been explained.

## Data Availability

Data supporting the findings of this study are included in this article.
